# Candid and Fragile: A Case Report of Infective Endocarditis From Candida albicans and Bacteroides fragilis

**DOI:** 10.7759/cureus.35456

**Published:** 2023-02-25

**Authors:** Jackson B Troxel, William S Ogan, Grant R Conner

**Affiliations:** 1 Internal Medicine, NEA Baptist Memorial Hospital, Jonesboro, USA; 2 Division of Bioinformatics, Department of Medicine, UC (University of California) Health San Diego, San Diego, USA

**Keywords:** case report, bacteroides fragilis, candida albicans, echocardiography, infective endocarditis

## Abstract

Infective endocarditis (IE) remains a significant cause of mortality worldwide and reported cases are continuing to increase annually. We describe a case of a patient who would undergo coronary artery bypass grafting (CABG) with bioprosthetic aortic valve replacement complicated by postop gastrointestinal bleeding requiring partial colectomy with ileocolic anastomosis who would later present with fever, dyspnea, and persistently positive blood cultures who would be found to have tricuspid valve endocarditis from *Candida* and *Bacteroides* species that was successfully treated with a combination of surgical resection and antimicrobial therapy.

## Introduction

*Bacteroides fragilis* (B. fragilis) is an anaerobic gram-negative bacillus normally found in the small and large intestines, as well as the vagina. Thus, this organism is more commonly found to be a source of infection in its native areas, causing intra-abdominal and gynecologic infections. Due to its anaerobic nature, B. fragilis is difficult to isolate and culture. There have been numerous risk factors identified to predispose an individual to bacteremia from anaerobic bacteria such as cancer, immunosuppression, corticosteroid use, use of cytotoxic agents, splenectomy, diabetes mellitus, and gynecological/gastrointestinal surgeries [1]. Few cases have been documented in the literature describing B. fragilis as the source of endocarditis, likely due to the aforementioned factors of its natural colonization locations as well as being difficult to grow in the laboratory.

## Case presentation

A 76-year-old female, with a significant history of coronary artery disease with recent coronary artery bypass grafting (CABG) and bioprosthetic aortic valve replacement, hypertension, and type 2 diabetes mellitus, initially presented with a gastrointestinal bleed and was found to have an ulcerated cecum and a fistula between a pancreatic pseudocyst and the hepatic flexure. She underwent a partial colectomy with ileocolic anastomosis and internal drainage of a pancreatic pseudocyst using a Roux-en-Y limb of the jejunum. Several days postop, she developed a wound infection that grew *Bacteroides fragilis* (B. fragilis) and was treated with appropriate antibiotics. Seven months later, she returned with chills and malaise and was found to be septic with B. fragilis bacteremia and was discharged on a prolonged course of ertapenem.

She returned to the ED three months later with chills, dyspnea on exertion, and fever and was admitted for further workup. Physical examination was notable for bilateral lower extremity edema with a 2/6 high-pitched, holosystolic murmur best heard at the lower right sternal border. A laboratory survey noted a leukocytosis of 15.7, with a neutrophil predominance, and elevated C-reactive peptide and erythrocyte sedimentation rate. Blood cultures were obtained on admission and vancomycin and piperacillin-tazobactam were started.

**Figure 1:**
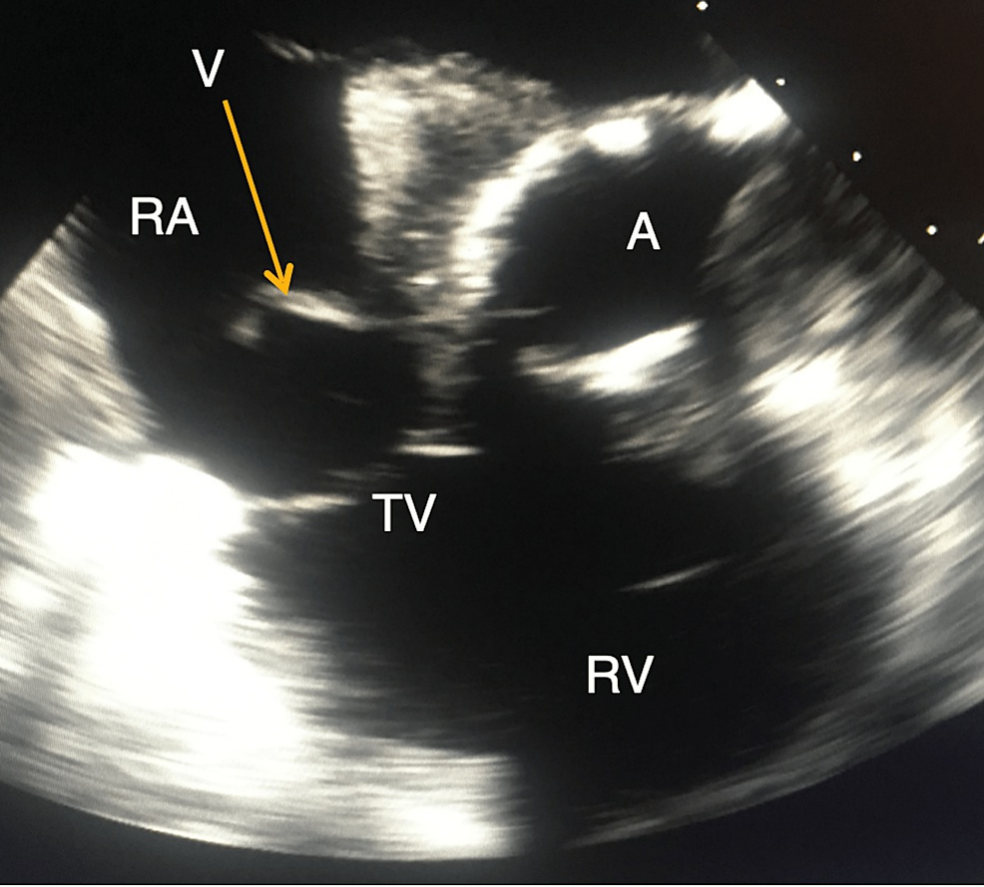
Transesophageal echocardiogram (TEE) TEE showing vegetation. (A = Aorta, RA = Right Atrium, RV = Right Ventricle, TV = Tricuspid Valve, V = Vegetation)

**Figure 2:**
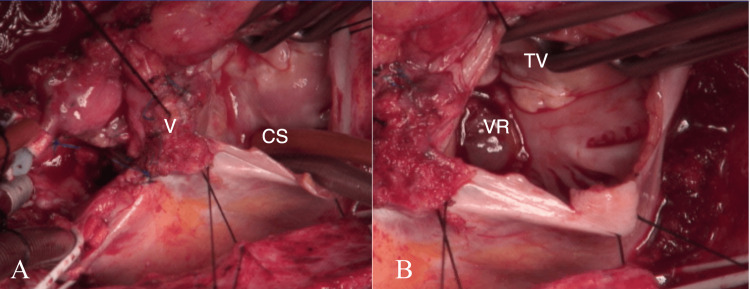
A: Intraoperative view of vegetation; B: Post-excision and debridement 2A: Surgical view into the right atrium (RA) with vegetation. (CS = Coronary Sinus, V = Vegetation) 2B: Same view as 2A but with vegetation removed. (TV = Tricuspid Valve, VR = Vegetation Removed)

Blood cultures would quickly grow B. fragilis and antibiotic therapy was changed to meropenem. Blood cultures would be drawn every 48 hours and continue to be positive with B. fragilis and would also start growing *Candida albicans* (C. albicans), prompting the addition of amphotericin B to the meropenem. Computed tomography of the abdomen and pelvis would not reveal any acute abnormalities or source of infection. In further pursuit of a source and due to the new onset murmur, a transthoracic echocardiogram (TTE) was obtained, which did not definitively identify vegetation. This was followed by a transesophageal echocardiogram (TEE) that revealed a mobile mass attached to the atrial side of the medial tricuspid annulus with thickening and a probable abscess extending into the tissue adjacent to the posterior medial aortic valve ring and atrial septum with the bioprosthetic aortic valve functioning normally (Figure 1). The Infectious Disease and Cardiothoracic Surgery services were consulted for assistance and, ultimately, it was decided to proceed with surgical treatment. The patient underwent re-do sternotomy with excision and debridement of the large right atrial vegetation, debridement of a right atrial septal phlegmon, tricuspid valve repair, and closure of a patent foramen ovale (Figures 2A-2B).

A smear of the specimen revealed budding yeast, but the culture noted no bacterial or fungal growth. Postoperatively, the patient was continued on intravenous antimicrobials until blood cultures were persistently negative and transitioned to oral fluconazole for six weeks following surgery.

## Discussion

Infective endocarditis (IE) is defined as an infection of a native or prosthetic heart valve, the endocardial surface, or an indwelling cardiac device [2]. The estimated incidence of IE is five to seven cases per 100,000 persons/year and, despite advancements in diagnosis and treatment, has a global mortality rate of approximately 25% [3]. Overall, *Staphylococcus aureus* is the most common cause of IE and is responsible for 31% of cases [4]. IE secondary to anaerobic bacteria, such as *Bacteroides,* is a rarer occurrence and has a mortality rate of 21-43% while IE from *Candida* species is rarely seen in immunocompetent individuals and most cases are only able to be definitively diagnosed post-mortem [5-7]. The mortality rate associated with IE due to *Candida* species is as high as 46.6-50.0%, making timely diagnosis and treatment of paramount importance [7]. While the most significant risk factor for *Candida* endocarditis is intravenous drug use, we believe the etiology, in this case, is the combination of multiple chronic indwelling central venous catheters and prolonged antibiotic exposure [8].

The modified Duke criteria were utilized in this case and aided in workup and diagnosis. In this case, the patient met the criteria for definitive IE based on the presence of two major criteria: persistently positive blood cultures from an organism known to cause endocarditis and echocardiographic findings of intracardiac vegetation (Table 1) [9].

**Table 1:** Modified Duke criteria for the diagnosis of infective endocarditis Adapted from Otto CM, Nishimura RA, Bonow RO, Carabello BA, Erwin JP, Gentile F, Jneid H, Krieger EV, Mack M, McLeod C, O’Gara PT, Rigolin VH, Sundt TM, Thompson A, Toly C [9]. Definite diagnosis of IE: 2 major criteria; or 1 major criterion + 3 minor criteria; or 5 minor criteria Possible diagnosis of IE: 1 major criterion + 1 minor criterion; or 3 minor criteria Rejected diagnosis of IE: Absence of criteria for definitive or possible IE

Modified Duke criteria for diagnosing infective endocarditis
Major Criteria
1. Positive blood culture
- Blood culture positive with typical microorganisms consistent with IE (Staphylococcus aureus, viridans streptococci, Streptococcus gallolyticus, HACEK (Haemophilus species, Aggregatibacter (formerly Actinobacillus) species, Cardiobacterium species, Eikenella corrodens, and Kingella species), and community-acquired Enterococci in the absence of a primary focus) from 2 separate blood cultures; or
- Microorganisms consistent with IE from persistently positive blood cultures (2 or more positive blood cultures of blood samples drawn > 12 h apart; or all of 3 or a majority of 4 or more separate cultures of blood (with first and last sample drawn at least 1 h apart); or
- Single positive blood culture for Coxiella burnetii or antiphase I IgG antibody titer ≥ 1:800
2. Evidence of endocardial involvement:
- Echocardiogram positive for IE (vegetation, abscess, pseudoaneurysm, intracardiac fistula, valvular perforation or aneurysm, or new partial dehiscence of prosthetic valve)
- New valvular regurgitation
Minor Criteria
1. Predisposition (predisposing heart condition or injection drug use)
2. Fever (temperature > 38°C)
3. Vascular phenomena, major arterial emboli, septic pulmonary infarcts, mycotic aneurysm, intracranial hemorrhage, conjunctival hemorrhages, and Janeway lesions
4. Immunologic phenomena: glomerulonephritis, Osler nodes, Roth’s spots, and rheumatoid factor
5. Microbiological evidence: positive blood culture but does not meet major criterion or serologic evidence of active infection with organisms consistent with IE

Intravenous antimicrobials remain the mainstay of treatment of IE, and the choice of agent and duration of therapy varies depending on the organisms identified and if treating native valve endocarditis (NVE) or prosthetic valve endocarditis (PVE). The duration of antimicrobial therapy in NVE is typically four weeks while the duration in PVE is typically six weeks [9]. Surgical intervention is indicated in specific instances and if the valve is right-sided, left-sided, or a prosthetic valve. For right-sided NVE, surgery is indicated for vegetations (≥20 mm in diameter), recurrent septic pulmonary emboli, highly resistant organisms, or persistent bacteremia [9]. For left-sided NVE, surgery is usually required earlier than in right-sided due to complications such as IE-associated valve dysfunction complicated by HF, intracardiac abscess, difficult-to-treat pathogen, and/or persistent infection [9]. Indications for surgical intervention in PVE are similar to that of NVE. In cases of *Candida* endocarditis, a combined treatment of antifungal therapy and valve repair/replacement is recommended by both the Infectious Disease Society of America and the American Heart Association [9,10].

## Conclusions

IE from anaerobic bacteria, such as *Bacteroides,* and from *Candida* species are rare occurrences on their own but even more so concomitantly. This case demonstrates a rare cause of endocarditis that was treated to cover two culprits: B. fragilis and C. albicans. The combination of fever, heart failure symptoms, and a new murmur in a patient with recent surgery involving the GI tract greatly increased the index of suspicion for infective endocarditis in this patient with persistently positive blood cultures from a bacterium known to inhabit the GI tract. Another interesting aspect of this case is that the bioprosthetic aortic valve was not involved and only the native tricuspid valve was affected. A literature review for cases documenting endocarditis from B. fragilis yields limited results and a documented case of endocarditis from both B. fragilis and C. albicans could not be found. Due to this, we believe this to be one of the first case reports of a patient with IE caused by B. fragilis and C. albicans, and the non-ubiquitous nature of this type of endocarditis warrants its documentation in the literature to observe how the context and comorbidities of the patient influence treatment outcomes.
